# Supplementary material for: Studying the Impact of Persistent Organic Pollutants Exposure on Human Health by Proteomic Analysis: A Systematic Review
Source: Int J Mol Sci. 2022 Nov 17;23(22):14271. doi: 10.3390/ijms232214271 (PMC9692675; doi:10.3390/ijms232214271)
Supplement: Supplementary file 1 [file ijms-23-14271-s001.zip › ijms-2028161-supplementary.pdf]

Supplementary Materials: Search strategy (Pubmed)

1) aldrin proteomic
2) ("Aldrin" [Mesh]) AND "proteomics" [Mesh]
3) chlordane proteomic
4) (“Chlordan" [Mesh]) AND "proteomics" [Mesh]
5) chlordecone proteomic
6) ("Chlordecone" [Mesh]) AND "proteomics" [Mesh]
7) decabromodiphenyl ether proteomic
8) BDE-209 proteomic
9) ("Decabromodiphenyl ether" [Mesh]) AND "proteomics" [Mesh]
10) dicofol proteomic
11) ("Dicofol" [Mesh]) AND "proteomics" [Mesh]
12) dieldrin proteomic
13) ("Dieldrin" [Mesh]) AND "proteomics" [Mesh]
14) endrin proteomic
15) ("Endrin" [Mesh]) AND "proteomics" [Mesh]
16) heptachlor proteomic
17) ("Heptachlor" [Mesh]) AND proteomics [Mesh]
18) hexachlorobenzene proteomic
19) HCB proteomic
20) ("Hexachlorobenzene" [Mesh]) AND "proteomics" [Mesh]
21) hexachlorobutadiene proteomic
22) ("Hexachlorobutadiene" [Mesh]) AND "proteomics" [Mesh]
23) alpha hexachlorocyclohexane proteomic
24) ("Alpha hexachlorocyclohexane" [Mesh)] AND "proteomics" [Mesh]
25) beta hexachlorocyclohexane proteomic
26) ("Beta hexachlorocyclohexane" [Mesh]) AND "proteomics" [Mesh]
27) lindane proteomic
28) gamma hexachlorocyclohexane proteomic
29) ("Hexachlorocyclohexane" [Mesh]) AND "proteomics" [Mesh]
30) mirex proteomic
31) ("Mirex" [Mesh]) AND "proteomics" [Mesh]
32) pentachlorobenzene proteomic
33) PeCB proteomic
34) ("Pentachlorobenzene" [Mesh]) AND "proteomics" [Mesh]
35) pentachlorophenol proteomic
36) ("Pentachlorophenol" [Mesh]) AND "proteomics" [Mesh]
37) polychlorinated biphenyl proteomic
38) PCB proteomic
39) ("Polychlorinated biphenyls" [Mesh]) AND "proteomics" [Mesh]
40) polychlorinated naphtalenes proteomic
41) "polychlorinated naphtalenes" AND "proteomics" [Mesh]
42) PFOA proteomic
43) perfluorooctanoic acid proteomic
44) "perfluorooctanoic acid" AND "proteomics" [Mesh]
45) hexabromobiphenyl proteomic
46) ("Polybrominated biphenyls" [Mesh]) AND "proteomics" [Mesh]
47) hexabromocyclododecane proteomic
48) HBCD proteomic
49) ("Hexabromocyclododecane" [Mesh]) AND "proteomics" [Mesh]
50) hexabromodiphenyl ether proteomic

51) ("Hexabromodiphenyl ether 154" [Mesh]) AND "proteomics" [Mesh]
52) heptabromodiphenyl ether proteomic
53) ("2,2',3,4,4',5',6-heptabromodiphenyl ether" [Mesh]) AND "proteomics [Mesh]
54) short-chain chlorinated paraffins proteomic
55) SCCPs proteomic
56) "short-chain chlorinated paraffins" AND "proteomics" [Mesh]
57) endosulfan proteomic
58) ("Endosulfan" [Mesh]) AND "proteomics" [Mesh]
59) tetrabromodiphenyl ether proteomic
60) BDE-47 proteomic
61) ("2,2',4,4'-tetrabromodiphenyl ether" [Mesh]) OR ("2,2',4,5'-tetrabromodiphenyl ether" [Mesh]) AND "proteomics" [Mesh]
62) pentabromodiphenyl ether proteomic
63) BDE-99 proteomic
64) ("Pentabromodiphenyl ether" [Mesh]) AND "proteomics" [Mesh]
65) toxaphene proteomic
66) ("Toxaphene" [Mesh]) AND "proteomics" [Mesh]
67) DDT proteomic
68) dichlorodiphenyltrichloroethane proteomic
69) ("DDT" [Mesh]) AND "proteomics" [Mesh]
70) PFOS proteomic
71) perfluorooctane sulfonic acid proteomic
72) ("Perfluorooctane sulfonic acid" [Mesh]) AND "proteomics" [Mesh]
73) perfluorooctane sulfonyl fluoride proteomic
74) "perfluorooctane sulfonyl fluoride" AND "proteomics" [Mesh]
75) hexachlorobenzene proteomic
76) HCB proteomic
77) ("Hexachlorobenzene" [Mesh]) AND "proteomics" [Mesh]
78) hexachlorobutadiene proteomic
79) HCBD proteomic
80) ("Hexachlorobutadiene" [Mesh]) AND "proteomics" [Mesh]
81) pentachlorobenzene proteomic
82) PeCB proteomic
83) ("Pentachlorobenzene" [Mesh]) AND "proteomics" [Mesh]
84) polychlorinated dibenzo-p-dioxins proteomic
85) PCDD proteomic
86) ("Polychlorinated dibenzodioxins" [Mesh]) AND "proteomics" [Mesh]
87) polychlorinated dibenzofurans proteomic
88) PCDF proteomic
89) ("Dibenzofurans, polychlorinated" [Mesh]) AND "proteomics" [Mesh]
90) polychlorinated naphtalenes proteomic
91) PCN proteomic
92) "Polychlorinated naphtalenes" AND "proteomics" [Mesh]

No search filters were applied
Records identified in MEDLINE N = 749
